# Biochemical adaptations of four submerged macrophytes under combined exposure to hypoxia and hydrogen sulphide

**DOI:** 10.1371/journal.pone.0182691

**Published:** 2017-08-04

**Authors:** Mahfuza Parveen, Takashi Asaeda, Md H. Rashid

**Affiliations:** 1 Graduate School of Science and Engineering, Saitama University, 255 Shimo-okubo, Sakura-ku, Saitama, Japan; 2 Department of Environmental Science and Technology, Saitama University, 255 Shimo-okubo, Sakura-ku, Saitama, Japan; 3 Research Institute of Chuo University, Kasuga, Bunkyo, Tokyo, Japan; 4 Department of Agronomy, Bangladesh Agricultural University, Mymensingh, Bangladesh; Universidade de Lisboa Instituto Superior de Agronomia, PORTUGAL

## Abstract

A hydroponic experiment was performed to investigate the stress responses and biochemical adaptations of four submerged macrophytes, *Potamogeton crispus*, *Myriophyllum spicatum*, *Egeria densa*, and *Potamogeton oxyphyllus*, to the combined exposure of hypoxia and hydrogen sulfide (H_2_S, provided by NaHS). The investigated plants were subjected to a control, hypoxia, 0.1mM NaHS, 0.5 mM NaHS, 0.1 mM NaHS+hypoxia and 0.5 mM NaHS+hypoxia conditions. All experimental plants grew optimally under control, hypoxic and NaHS conditions in comparison to that grown in the combined exposure of hypoxia and hydrogen sulfide. For *P*. *crispus* and *M*. *spicatum*, significant decreases of total chlorophyll and increases in oxidative stress (measured by hydrogen peroxide, H_2_O_2_, and malondialdehyde, MDA) were observed with exposure to both sulfide concentrations. However, the decrease in catalase (CAT) and ascorbate peroxidase (APX) from exposure to 0.5 mM NaHS suggests that the function of the protective enzymes reached their limit under these conditions. In contrast, for *E*. *densa* and *P*. *oxyphyllus*, the higher activities of the three antioxidative enzymes and their anaerobic respiration abilities (ADH activity) resulted in higher tolerance and susceptibility under high sulfide concentrations.

## Introduction

Knowledge regarding submerged macrophytes and environmental factors is essential for understanding aquatic plant ecophysiology and ecosystem productivity. Submerged macrophytes are one of the key components in aquatic ecosystems and play an important role as primary producers. Any negative effects on them can hinder the viability of the aquatic ecosystem. The distribution of submerged macrophytes is dependent on several biotic and abiotic factors such as sediment anoxia [1], water column hypoxia [2,3], water movement [4,5], nutrient availability in both the sediment and water column [6–8], light availability [9], heavy metals, pH and temperatures. Among them, dissolved oxygen is one of the important environmental factors during the life cycle of submerged macrophytes. In fresh water and coastal marine ecosystems, dissolved oxygen (DO) can drastically change compared to other environmental factors such as flooding, stagnation and eutrophication.

Hypoxia may not act as a stressor alone, and it can co-occur in synergy with other stressors, such as hydrogen sulfide (a common toxic product of anoxic sediment). The pH of the water can have a strong influence on the chemical speciation of sulfide (H_2_S, HS^-^ and S^2-^). Although all forms seem to be equally toxic [10], the gaseous H_2_S will normally prevail over both ionic forms in freshwater systems. Because the pH of most anaerobic soils is buffered at approximately 6–7 as a result of the HCO_3_^-^ - CO_2_ buffering mechanism, relative H_2_S abundance is approximately 60–95% [11]. H_2_S is produced as a metabolic end product by microbially mediated organic matter decomposition and dissimilatory sulfate reduction in waterlogged soil. Regarding the aquatic plant responses to sulfide exposure, sulfide tolerance in sea grass species is relatively high (2000–6000 μM L^-1^) [11]. Moreover, larger halophytes show tolerance to high sulfide concentrations (500–1500 μM L^-1^) compare to other aquatic macrophytes (10–500 μM L^-1^). Toxicity effects were reported for *E*. *nuttallii*, *P*. *compressus*, and *H*. *verticillata* when they were exposed to 100–600 μM L^-1^ sulfide concentrations [12,13]. For the present study, two concentrations of H_2_S supplied by NaHS (0.1 and 0.5 mM) were selected based on previously cited literature and laboratory experiments. By observing differences in sulfide tolerance between species in the literature, it can be hypothesized that the enhancement of plant growth and tolerance to various H_2_S concentrations is species specific.

Hydrogen peroxide (H_2_O_2_) is a common reactive oxygen species (ROS) formed continuously as a by-product of the regular metabolism of oxygen. However, H_2_O_2_ levels can increase dramatically under stress conditions, which can cause damage in cells and tissues and seriously disrupt metabolism via the oxidation of membrane lipids, proteins, pigments and nucleic acids. [14]. To overcome this situation, cells are equipped with enzymatic and non-enzymatic mechanisms to eliminate or reduce their damaging effects [15]. Moreover, an effective antioxidant system is vital for keeping intracellular ROS pools at low levels and for processing ROS effectively [16]. The enzymatic antioxidants include superoxide dismutase (SOD), catalase (CAT), guaiacol peroxidase (POD), glutathione peroxidase (GPx), and glutathione *S*-transferase (GST) and ascorbate peroxidase (APX). Submerged macrophytes have certain physiological adaptations to hypoxia and sulfide toxicity, such as antioxidative enzymes increment [17,18] and anaerobic respiration [19]. Alcohol dehydrogenase activity (ADH) is an active anaerobic fermentation enzyme that catalyses the terminal step in alcoholic fermentation [20] and is synthesized favourably under low O_2_ concentrations. Pyruvate is the end product of glycolysis that is converted to acetaldehyde, which is further converted to ethanol by ADH to generate NAD^+^ from NADH [21]. Therefore, pyruvate and ADH activity in plants subjected to sulfide and low oxygen stress appears to be an adaptation for anoxia tolerance.

The effects of hypoxia and sulfide on aquatic plants have been extensively studied [13,22,23], and only a few studies have determined the effects of hypoxia and dissolved H_2_S on submerged macrophytes. The effects of sediment anoxia on submerged macrophytes was evaluated by Zaman and Asaeda [1]. However, the study did not evaluate the effects of sulfide on submerged macrophytes under hypoxia. Therefore, the present study investigated the combined effects of water column hypoxia and exogenous H_2_S concentration on the biochemical adaptations of four submerged macrophyte species: *Potamogeton crispus*, *Myriophyllum spicatum*, *Egeria densa*, and *Potamogeton oxyphyllus*. These are cosmopolitan species and occur abundantly in Japan and the rest of the world.

## Methods

### Plant samples and experimental setup

We used data generated from laboratory experiments for the present study. Plants were collected from the rivers for planting and culturing in the tanks where no specific permission was required. We didn’t involve any endangered or protected species in any stage of the study. Plant samples of *M*. *spicatum* and *P*. *crispus* were collected from the Moto-Arakawa River, a tributary of the Arakawa River in southern Saitama, Japan (36° 7ʹ 30.1ʺ N, 139° 24ʹ 20ʺ E), and *E*. *densa* and *P*. *oxyphyllus* were collected from the Hofu River, Hiroshima (34° 11’ 390 ʺ N, 131.39’249ʺ E) and the Hii River, Shimane (35° 19´ 52.3ʺ N 132° 46´ 8.8ʺE), Japan, respectively. After collection, they were transported to the laboratory as early as possible and immediately cultured in a growth chamber at a controlled temperature of 23±3°C and a 12:12 (light:dark) photoperiod. The light intensity was maintained at approximately 100 μM m^-2^ s^-1^ by using fluorescent lamp tubes. Commercial river sand (DIY, Doite, Japan, 90% <1 mm particle) was used as the substrate. The experimental plants were obtained from these culture tanks. After one month of acclimation, two apical tips (~6 cm) were clipped and plugged into silicone sponge clumps and placed in a 500 ml glass beaker. The culture medium was 5% Hoagland’s nutrient solution (HNS) [24]. In total six treatments, control, hypoxia, 0.1mM NaHS, 0.5 mM NaHS, 0.1 mM NaHS+hypoxia and 0.5 mM NaHS+hypoxia were selected for each plant, with three replicates.For the hypoxic treatment, the beaker was placed in a 2.5 L AnaeroJar (Oxoid AG25, Oxoid Ltd., Basingstoke, England) [25] after deoxygenating the water by bubbling with anaerobic gas (a mixture of 9.38% CO_2_, 10.03% H_2_ with balanced N_2_). The oxygen concentration inside the jar was reduced with AnaeroPack (an atmospheric gas generating system, Mitsubishi Company, Japan), which can reduce the oxygen level by generating 10–15% CO_2_. Anaerobic indicators were used to check the low oxygen level (<0.1%) inside the jar. Such low oxygen and high CO_2_ under anoxia is a common phenomenon in natural conditions. For the H_2_S treatment, sodium hydrogen sulfide (NaHS) was used as a hydrogen sulfide (H_2_S) donor [26–29]. For the combination of H_2_S and hypoxia, the beaker was placed in an AnaeroJar and the medium was deoxygenated with anaerobic gas (same as hypoxia), followed by an application of NaHS to achieve the desired H_2_S concentrations. The lid of the AnaeroJar closed immediately just after the application of NaHS. The culture medium of each treatment was renewed after 24 hours due to the relatively short half-life of H_2_S [30]. The experiment was conducted for 3 days as plants exposed to hypoxia+H_2_S showed brown discoloration. The pH of the solution was maintained at 5.0 to 5.5 using NaOH or HCl for every treatment.

### Dissolved H_2_S and DO measurements in the water

Dissolved H_2_S was determined colorimetrically by the methylene blue method [31] using a diamine reagent. Four (4) ml of mixed diamine reagent was reacted with 50 ml water samples, and the amount of absorbance was measured spectrophotometrically at 670 nm after 20 minutes. NaHS was used as a calibration standard, and the results were expressed in mM. Dissolved oxygen (DO) was measured using a dissolved oxygen and temperature meter (HI 9146) and expressed as mg L^-1^.

### Determination of chlorophyll, IAA, H_2_O_2_, POD, APX and CAT via assays

The chlorophyll (total chl.) content was determined spectrophotometrically by extracting fresh shoots in 5 ml of N,N-dimethylformamide for 24 h in the dark at 4°C [32] and calculated using the equations of Porra et al. [33]; chlorophyll content was expressed as mg g^-1^ FW.

The concentration of indole acetic acid (IAA), the most abundant form of auxin in plant tissues, was measured using the Salkowski reagent [34]. Approximately 100 mg of fresh weight (FW) plant tissue from the apical tip was ground in 2.5 ml of distilled water and centrifuged at 5,000 × g at 20°C for 15 min. After collecting the supernatant, 1 ml of the extract was added to 2 ml of the Salkowski reagent, and colour development was measured after 1 hr at 530 nm [5]. The results were presented as μg g^-1^ FW.

For H_2_O_2_, POD, APX and CAT assays, approximately 100 mg of fresh plant shoots were extracted in ice-cold phosphate buffer (50 mM, pH 6.0) that contained polyvinylpyrrolidone (PVP). The extractions were centrifuged at 5,000 × g for 20 min at 4°C. The supernatant was collected and immediately stored at -80°C for further analysis.

For the analysis of the endogenous H_2_O_2_ concentration, a 750 μl aliquot was mixed with 2.5 ml of 0.1% titanium sulfate in 20% (v/v) H_2_SO_4_ [35]. The mixture was centrifuged at 5,000 × g at 20°C for 15 min. The intensity of the yellow colour was measured spectrophotometrically at 410 nm. H_2_O_2_ concentrations were estimated using a standard curve prepared from known concentrations of H_2_O_2_. The results were presented as μmol g^-1^ FW. POD (EC 1.11.1.7) was assayed according to the method of Goel et al. [36]. The change in absorbance was recorded at 470 nm in 15 s intervals for 3 min using an extinction coefficient of 26.6 mM^-1^ cm^-1^. APX (EC 1.11.1.11) activity was assayed using the methods described by Nakano and Asada [37]. The decrease in absorbance at 290 nm was recorded at every 15 s, and APX activity was determined using the extinction coefficient of 2.8 mM^-1^ cm^-1^_._ CAT activity (EC 1.11.1.6) was determined following the methods of Aebi [38] and was calculated using the extinction coefficient of 40 mM^-1^ cm^-1^. APX, POD and CAT activities were presented in μmol min^-1^ g^-1^ FW.

### Determination of MDA

The level of lipid peroxidation was measured in terms of malondialdehyde (MDA), a product of lipid peroxidation, in plant samples using a thiobarbituric acid (TBA) reaction according to the formula developed by Heath and Packer [39]. Absorbance was measured at 532 and 600 nm where the molar extinction coefficient for MDA was 155 mM^-1^ cm^-1^. The results were presented as nmol g^-1^ FW.

### ADH activity and pyruvate content

Alcohol dehydrogenase (ADH) activity (EC 1.1.1.1) was extracted from shoot samples using the methodology described by John and Greenway [40]. Briefly, 50 mg of shoot tissue was ground in liquid nitrogen, and cold extraction buffer was added at 5 ml g^− 1^. The ADH extraction buffer was 50 mM HEPES (4-2-hydroxyethylpiperazine-1-ethanesulfonic acid) (pH 8.0) containing 5 mM MgCl_2_, 2 mM cysteine hydrochloride and 2% w/v PVP-40 (polyvinylpyrrolidone, MW ≈ 40,000). The samples were homogenized with a mortar and pestle and centrifuged at 10,000×g at 4°C for 10 min. From the collected supernatant, 0.1 ml enzyme extract was assayed in the presence of 80 μM NADH and 10 mM acetaldehyde in a buffer solution of 40 mM bicine and 5 mM MgCl_2_ (pH 8.0) [41]. The decrease in absorbance was monitored at 340 nm, and the enzyme activity was calculated using an extinction coefficient of 6.22 mM^-1^ cm^-1^ [42]. The ADH activities were presented as μmol min^-1^ g^-1^ FW.

The pyruvate content in the plant shoots was determined using the 2,4-dinitrophenylhydrazine method [43]. Approximately 50 mg of apical tip tissue was frozen with liquid N_2_, ground with 2.25 ml 8% trichloroacetic acid (TCA), and centrifuged at 4900×g at 4°C for 10 min. The supernatant was collected, and a 1 ml aliquot was mixed with 2 ml 8% TCA, 1 ml 0.1% 2,4 dinitrophenylhydrazine and 5 ml 1.5 M NaOH. The pyruvate concentration was calculated from the standard curve generated with known concentrations of sodium pyruvate [44] and expressed as μmol g^-1^ FW.

### Statistical analyses

All experimental data were presented as the means ± SD (n = 3). The data were checked for normality before performing the statistical analysis. All data were subjected to a one-way analysis of variance (one-way ANOVA), followed by Tukey’s multiple comparison test to evaluate the mean differences at a 0.05 significance level (p<0.05). Pearson’s correlations were calculated among chlorophyll content, IAA, antioxidative enzymes, ADH activity, pyruvate content, MDA and H_2_O_2_. Statistical analyses were performed using SPSS for Windows (Release 17, SPSS INC., Chicago, IL).

## Results

**Fig 1:**
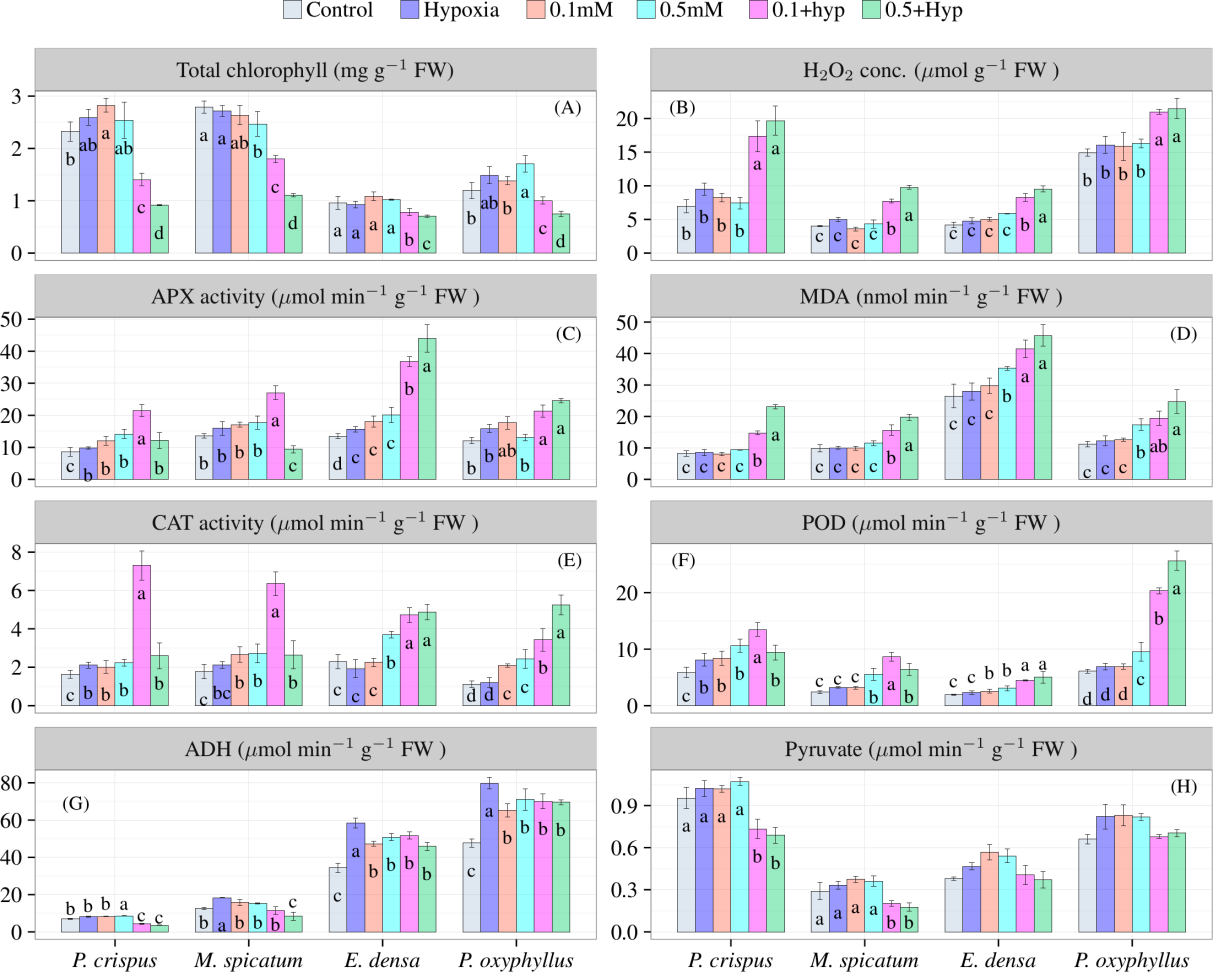
Effects of hypoxia and NaHS on total chlorophyll (A), H_2_O_2_ (B), APX activity (C), MDA (D), CAT activity (E), POD activity (F), ADH activity (G) and pyruvate content (H) values in *P*. *crispus*, *M*. *spicatum*, *E*. *densa* and *P*. *oxyphyllus*. Values are the means of three replicates±SD. Bars with different letters are significantly different at P<0.05.

In Fig 1(A), total chl. concentrations were varied among all four plants and treatments. For *P*. *crispus* and *M*. *spicatum*, total Chl. concentrations decreased significantly (P<0.05) when subjected to 0.1 and 0.5 mM NaHS+Hyp conditions. In contrast, the concentration was not significantly decreased for *E*. *densa* and *P*. *oxyphyllus* when plants were subjected to the first five treatments, although significant differences (P<0.05) were observed in 0.5 mM conditions. H_2_O_2_ and MDA content also increased significantly in the 0.1 and 0.5 mM NaHS+Hyp treatments, regardless of the species (Fig 1(B) and 1(C)). For *P*. *crispus* and *M*. *spicatum*, antioxidative enzymes (APX and CAT activity) significantly increased (P<0.05) in 0.1 mM NaHS+Hyp conditions, although they decreased in 0.5 mM NaHS+Hyp conditions (Fig 1(D) and 1(E)). Moreover, POD activity increased significantly (P<0.05) under both sulfide conditions (Fig 1(F)). For *E*. *densa* and *P*. *oxyphyllus*, antioxidative enzymes (APX, CAT and POD activity) significantly increased (P<0.05) in both the Hyp+ 0.1 and 0.5 mM NaHS treatments (Fig 1(D), 1(E) and 1(F)). No significant differences were observed for any of the studied parameters when plants exposed to hypoxic conditions were compared to the control. ADH activity increased significantly (P<0.05) under hypoxic conditions (Fig 1(G)). The increase was significantly different among the four studied plants (*P*. *crispus*, 17%; *M*. *spicatum*, 45%; *E*. *densa*, 70%; and *P*. *oxyphyllus*, 68%). In the 0.1 and 0.5 mM NaHS+Hyp treatments, ADH activity increased for *E*. *densa* (50% and 30%, respectively) and *P*. *oxyphyllus* (47% and 46%, respectively); however, ADH activity decreased for *P*. *crispus* (-9% and -30%, respectively) and *M*. *spicatum* (-8% and -16%, respectively). Likewise, pyruvate content for *P*. *crispus* and *M*. *spicatum* decreased significantly (P<0.05) in both treatments (Fig 1(H)); in contrast, the differences were not significant among the treatments for the remaining two species.

**Fig 2:**
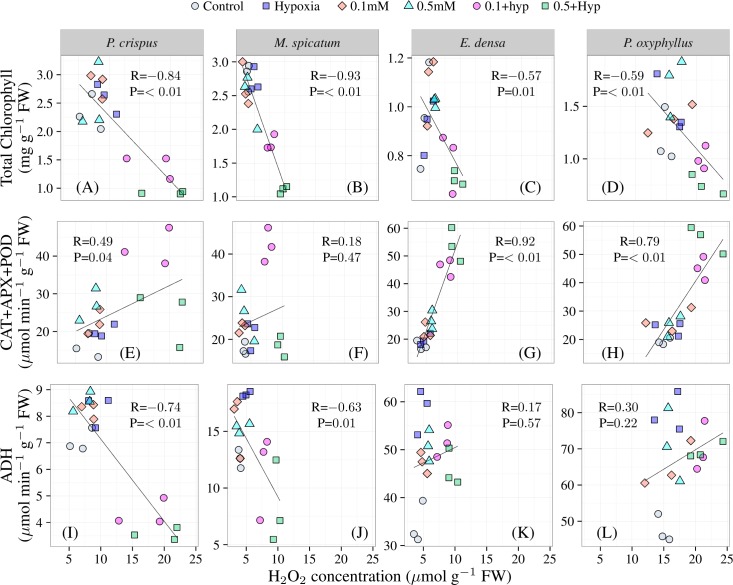
The correlations between H_2_O_2_ and total chlorophyll (A-D), H_2_O_2_ and antioxidative enzymes (CAT+APX+POD) (E-H), and H_2_O_2_ and ADH activity (I-L) in four plants exposed to different treatments.

Fig 2 shows the correlations between H_2_O_2_ and total chlorophyll (Chl.) (Fig 2A to 2D), H_2_O_2_ and antioxidative enzymes (CAT+APX+POD) (Fig 2E to 2H), and H_2_O_2_ and ADH activity (Fig 2I to 2L) in four plants exposed to different treatments. Strong positive correlations were observed between the ADH activity and H_2_O_2_ concentrations, and antioxidative enzymes and H_2_O_2_ concentrations in *P*. *oxyphyllus* and *E*. *densa* compare to *P*. *crispus* and *M*. *spicatum*. Total Chl. contents were negatively correlated to H_2_O_2_ concentration for all plants (Fig 2A to 2D). Positive correlations were observed for antioxidative enzymes (CAT, APX, POD) and H_2_O_2_ concentration, irrespective of plant species (Fig 2E to 2H). However, the correlations were highly significant (P<0.01) for *E*. *densa* and *P*. *oxyphyllus*, significant for *P*. *crispus* (P<0.05) and not significant for *M*. *spicatum* (P<0.47). Significant negative correlations were observed between ADH activity and H_2_O_2_ concentration for *P*. *crispus* (R = -0.74) and *M*. *spicatum* (R = -0.63), and it was positive (not significant) for *E*. *densa* (R = 0.17) and *P*. *oxyphyllus* (R = 0.3).

The dissolved oxygen (DO) and H_2_S concentrations of experimental tanks were measured at the beginning and after 24 hours of the experiment (Table 1). For H_2_S+hypoxia experiment NaHS can produce desired amount of H_2_S at the beginning of the experiment, but decreased after 24 hours. To keep the desired H_2_S concentrations the media of every experiment changed after 24 hours. In Table 2 the ANOVA results (P and F values) of every treatment were listed.

**Table 1:** Dissolved oxygen (DO, mg L^-1^) and dissolved H_2_S concentrations (mM) in different treatments measured initially and after 24 hours. Values are the means of three replicates (n = 3).

	*P*. *crispus*				*M*. *spicatum*				*E*. *densa*				*P*. *oxyphyllus*			
	Initial		After 24 hours		Initial		After 24 hours		Initial		After 24 hours		Initial		After 24 hours	
	DO	H_2_S	DO	H_2_S	DO	H_2_S	DO	H_2_S	DO	H_2_S	DO	H_2_S	DO	H_2_S	DO	H_2_S
Control	6.26	0.00	6.50	0.00	6.37	0.00	6.30	0.00	6.34	0.00	6.50	0.00	6.34	0.00	6.50	0.00
Hypoxia	0.00	0.00	0.85	0.00	0.00	0.00	0.91	0.00	0.00	0.00	0.81	0.00	0.00	0.00	0.76	0.00
0.1mM	6.19	0.00	5.65	0.00	6.26	0.00	5.65	0.00	6.30	0.00	5.91	0.00	6.30	0.00	5.91	0.00
0.5mM	6.27	0.00	5.17	0.00	6.20	0.00	5.17	0.00	6.40	0.00	5.17	0.00	6.40	0.00	5.17	0.00
0.1+Hyp	0.00	0.09	0.00	0.07	0.00	0.09	0.00	0.06	0.00	0.09	0.00	0.05	0.00	0.09	0.00	0.05
0.5+Hyp	0.00	0.43	0.00	0.26	0.00	0.43	0.00	0.28	0.00	0.44	0.00	0.23	0.00	0.44	0.00	0.23

**Table 2:** ANOVA table for total chlorophyll (mg g^-1^ FW), H_2_O_2_ (μmol g^-1^ FW), APX (μmol min^-1^ g^-1^ FW), MDA (nmol min^-1^ g^-1^ FW), CAT (μmol min^-1^ g^-1^ FW), POD (μmol min^-1^ g^-1^ FW), ADH activity (μmol min^-1^ g^-1^ FW), pyruvate content (μmol min^-1^ g^-1^ FW) in *P*. *crispus*, *M*. *spicatum*, *E*. *densa* and *P*. *oxyphyllus* (n = 3).

	*P*. *crispus*		*M*. *spicatum*		*E*. *densa*		*P*. *oxyphyllys*	
	F-value (5, 12)	P-value	F-value (5, 12)	P-value	F-value (5, 12)	P-value	F-value (5, 12)	P-value
Total chlorophyll	16.76	<0.01	22.08	<0.01	3.81	0.03	8.18	<0.01
H_2_O_2_	14.25	<0.01	47.15	<0.01	28.28	<0.01	5.31	0.01
APX	7.87	<0.01	12.99	<0.01	31.63	<0.01	11.87	<0.01
MDA	71.33	<0.01	15.82	<0.01	7.78	<0.01	6.30	<0.01
CAT	22.32	<0.01	11.22	<0.01	13.74	<0.01	15.54	<0.01
POD	4.97	0.01	11.07	<0.01	6.63	<0.01	61.53	<0.01
ADH	79.74	<0.01	6.27	<0.01	13.93	<0.01	8.67	<0.01
Pyruvate	8.54	<0.01	5.62	0.01	2.95	0.06	2.42	0.10

## Discussion

The present study revealed that the four studied plants have the ability to survive in hypoxic conditions compared to the NaHS+Hyp conditions. Among them, *P*. *oxyphyllus* and *E*. *densa* have high tolerances to sulfide exposure compared to *P*. *crispus* and *M*. *spicatum*; additionally, the plants had different strategies in antioxidative responses and anaerobic respiration metabolism. Compared to *P*. *crispus* and *M*. *spicatum*, the higher antioxidant systems and anaerobic respiration abilities of *P*. *oxyphyllus* and *E*. *densa* stimulated their tolerances in high sulfide conditions.

The oxidative stress and the responses of antioxidative enzymes of the four submerged macrophytes were negatively affected by the presence of sulfide during water column hypoxia. Compared to control conditions, chlorophyll concentrations were reduced with increasing sulphide+hypoxia exposure for all study plants. The chlorophyll concentrations of *E*. *densa* and *P*. *oxyphyllus* were not significantly decreased at 0.1 mM NaHS+Hyp, which corroborates the findings of Dooley et al. [45] and Chen et al. [46] in an experiment with *Zostera marina* and *Spinacia oleracea* seedlings, respectively. Chloroplast biogenesis might be a partial reason for this phenomenon. Holmer and Bondgaard [47] also reported that chlorophyll a concentrations of eelgrass plant (*Z*. *marina*) decreased with increased sulfide concentrations under low oxygen exposure and different sulfide concentrations, thus demonstrating the consistency of the present study.

It was also visually observed that the investigated plants exposed to NaHS+Hyp conditions showed brown discoloration, which was quickly caused by chlorophyll degradation. This reduction can occur due to the accumulation of H_2_O_2_, given that chloroplast is one of the main organelles that produce ROS in plant cells. It is reported that H_2_O_2_ is a strong inhibitor of photosynthesis, which can inhibit CO_2_ fixation by 50% due to the oxidation of the thiol-modulated enzymes of the Calvin cycle [16].

Plants have evolved both enzymatic and non-enzymatic scavenging systems to mitigate the overproduction of ROS. In plants, catalase (CAT), ascorbate peroxidase (APX), guaiacol peroxidase (POD) are considered the most important H_2_O_2_ scavengers, and their increasing activities in plants indicate oxidative stress [48–50]. The present results suggested that the overproduction of H_2_O_2_ under the different stresses was due to a concomitant increase in POD, APX and CAT activities (Fig 1). APX and CAT belong to two different classes of H_2_O_2_ scavenging enzymes; APX is responsible for the fine modulation of ROSs for signalling, whereas CAT is responsible for the removal of excess ROSs during stress [51]. Moreover, H_2_O_2_ is detoxified to H_2_O and O_2_ through CAT activity or through the ascorbate-glutathione cycle via the activity of APX [51]. Antioxidant systems have evolved not to completely remove ROS but to allow these signals to persist within the cellular environment. Hence, high or enhanced antioxidant capacity can be considered beneficial because it desensitizes photosynthesis, and in some cases, enhances the water-water cycle activity. In the present study, CAT and APX activity decreased for *P*. *crispus* and *M*. *spicatum* when they were exposed to the 0.5 mM NaHS+Hyp treatment, which suggested that these two enzymes were not able to scavenge the overproduction of ROS in a high sulfide environment.

POD is an essential component for plants for growth and senescence processes and is considered a stress marker enzyme with a high affinity for H_2_O_2_ [1,52]. It is activated as a short-term stress response [53], affects lignin and ethylene synthesis and the decomposition of IAA, and is involved in resistance against pathogens and promotes wound healing [54]. The results showed that the activities of POD increased to scavenge H_2_O_2_ during sulfide exposure in all studied plants. For *M*. *spicatum* and *P*. *crispus*, POD showed higher activity compared to APX and CAT during exposure to 0.5+hypoxia conditions but was not enough to survive under the reported conditions. During the exposure to high sulfide concentrations, these two plants lost their intrinsic balance due to the disturbance of the membrane system, which was measured as MDA content. MDA is a cytotoxic product of lipid peroxidation and has widely been used as an indicator of free radical production and consequent tissue damage [55]. The experimental results showed that with increasing sulfide concentration, the MDA concentration increased, which had a positive correlation with H_2_O_2_. Because there is a threshold of enzyme activity, the protective function of the three enzymes to the membrane system is limited [56]. For *P*. *crispus* and *M*. *spicatum*, the activities of the two antioxidative enzymes were low at the high sulfide concentration (0.5 mM), suggesting that the functions of the protective enzymes reached their limit under these conditions. The data are supported by several previous studies that evaluated submerged macrophytes subjected to heavy metal stress [57–59]. In contrast, for *E*. *densa* and *P*. *oxyphyllus*, the higher activities of the three antioxidative enzymes resulted in higher tolerance and susceptibility in the high sulfide concentrations. In addition to the antioxidative enzyme pyruvate also have the ability to remove excess H_2_O_2_ from the cell [60].

Hypoxia and sulfide are two key environmental stresses found in a freshwater ecosystem. The anaerobic respiration of submerged macrophytes is an important survival mechanism in these stresses [21]. ADH activity catalyses the terminal step in anaerobic fermentation [41], which is necessary for a plant to survive in such conditions [42]. Maricle et al. [41] suggested that the ability to increase ADH activity is an adaptation of estuarine- and flooding-tolerant plants to tolerate their natural habitats, which also contain sulfide. In the present study, high ADH activity and pyruvate content were observed when plants were exposed to hypoxic conditions. This suggests a well-developed capacity of the studied plants to perform anaerobic respiration. A similar trend of ADH activity showed for two wetland macrophytes (*S*. *alterniflora* and *P*. *hemitomon*) under high sulfide exposure [20]. The ADH activity result is also consistent with several previous studies [21,42]. However, ADH activity is very sensitive to sulfide exposure. The increase in ADH activity of *P*. *oxyphyllus* and *E*. *densa* under sulfide exposure made them more tolerant than *P*. *crispus* and *M*. *spicatum*.

In plants, different sulfide tolerance mechanisms were discussed, which included mechanisms of avoiding sulfide exposure, oxidizing sulfide, or excluding sulfide from the body [61] and metabolic adaptations (cytochrome c oxidase and ADH activity) [62]. The present study suggests that the increase in antioxidative enzymes could be another possible mechanism for aquatic plants to become sulfide tolerant in high sulfide environments. This research provides an understanding of the distribution and habitat preferences of submerged macrophytes and can eventually be used as an ecosystem management tool.
